# Impact of treatment modalities on prognosis of patients with metastatic renal collecting duct carcinoma

**DOI:** 10.1038/s41598-022-16814-y

**Published:** 2022-07-25

**Authors:** Xiaoyuan Qian, Junlai Wan, Yuanzhong Tan, Zhenrui Liu, Ying Zhang

**Affiliations:** 1 Department of Urology, Tongji Hospital, Tongji Medical College, Huazhong University of Science and Technology, Wuhan, China; 2 Department of Orthopedics, Tongji Hospital, Tongji Medical College, Huazhong University of Science and Technology, Wuhan, China; 3 Department of Nephrology, Central Hospital of Tujia and Miao Autonomous Prefecture, Enshi, Enshi Prefecture China; 4 Department of Nephrology, Tongji Hospital, Tongji Medical College, Huazhong University of Science and Technology, 1095 Jiefang Avenue, Wuhan, 430030 Hubei Province China

**Keywords:** Urological cancer, Oncology

## Abstract

Although patients with renal collecting duct carcinoma (CDC) benefit from surgery, the value of cytoreductive nephrectomy (CNx) for the prognosis of patients with metastatic CDC remains unclear. Hence, in this study, we used data from Surveillance, Epidemiology, and End Results (SEER) registry to investigate the prognostic factors and the impact of CNx on the outcomes in patients with metastatic CDC. Data of 521 patients, diagnosed with CDC between 2000 and 2018, were retrieved from the SEER database. Kaplan–Meier method and log-rank tests were used to compare the survival differences between the CNx group and non-surgical group. Multivariate Cox regression analysis was used to identify the risk factors associated with overall survival (OS) and cancer-specific survival (CSS) for patients with metastatic CDC. Moreover, multivariate Cox regression analysis guided by directed acyclic graphs (DAG) was used to unfold the impact of CNx and chemotherapy on OS and CSS. 86 patients were identified to have metastatic CDC. The median OS and CSS time were 5 and 6 months, respectively. The OS rates at 1-, 2- and 5-years were 24.4%, 15.1% and 2.3%, respectively. Whereas, the CSS rates at 1-, 2- and 5-years were 27.0%, 17.9% and 2.8%, respectively. Old patients and those receiving CNx or chemotherapy exhibited better survival outcomes. The multivariate regression model identified non-surgical treatment as the only independent prognostic factor for both, OS and CSS. However, DAG-guided multivariate Cox regression model showed that both, CNx and chemotherapy, were associated with both, OS and CSS. Patients with metastatic CDC exhibited worse clinical outcomes. However, CNx improved the prognosis of patients with metastatic CDC. Additionally, surgical resection of visible lesions and suitable chemotherapy were identified as alternative treatment strategies.

## Introduction

Renal collecting duct carcinoma (CDC) originates from the distal segment of the renal medullary collecting duct^1–3^. It exhibits an invasive biological behavior, and is a rare subtype accounting for < 2% of all renal cell carcinoma (RCC) cases^4^. Big data analyses suggest that incidence of CDC is extremely low, with the overall age-adjusted incidence being 0.2990 per 1,000,000 population over the last decade^4^. To date, a large series of studies from the European, Japanese and SEER databases have revealed that patients with CDC exhibit invasive biological behavior and worse median survival when compared to those with RCC, and that patients with CDC can benefit from surgery and chemotherapy^4–8^. However, there are only a few relevant studies on metastatic CDC. Thus, clinicians have a poor understanding of this metastatic disease. Since metastatic diseases have a malignant clinical course, timely and reasonable intervention is often essential for improving their prognosis. Therefore, comprehensive studies on clinicopathologic characteristics, treatment options and survival outcomes of metastatic CDC are needed.

Surgery is often chosen for management of CDC based on established standard treatments for common renal tumors. Previous studies demonstrated that patients receiving surgery survive longer than those who do not undergo surgery for CDC^4,7^. However, metastatic features of the tumor may prevent this potential survival benefit obtained from surgical treatment. Hence, the application of cytoreductive nephrectomy (CNx) surgery for patients with metastatic CDC needs to be verified. Besides, based on histological similarities between CDC and urothelium carcinoma, chemotherapy was also used for management of patients with CDC^9,10^. However, the response rate of CDC to chemotherapy is limited.

Since metastatic CDC is rare, data on treatment course and outcomes of patients with metastatic CDC have not yet been fully determined. In this study, we use the SEER database to investigate the risk factors that affect the outcomes of patients with metastatic CDC. Additionally, we explore the value of surgery and chemotherapy for the treatment of patients with metastatic CDC by using directed acyclic graphs (DAG) guided-Cox regression models.

## Results

### Clinical characteristics of metastatic CDC

Based on the inclusion criteria, 86 patients with metastatic CDC were enrolled in this analysis. The baseline clinicopathological characteristics of these patients are summarized in Table 1. Of these patients, 25 (29.1%) were females and 61 (70.9%) were males. With regards to age, 57 (66.3%) patients were < 68-year-old, whereas 29 (33.7%) were > 68-year-old.

**Table1:** Clinicopathologic Characteristics of Patients with Metastatic CDC.

Variables	Number (%)
Age(years)	
< 68	57 (66.3%)
≥ 68	29 (33.7%)
Sex	
Female	25 (29.1%)
Male	61 (70.9%)
Race	
Black	19 (22.1%)
White	61 (70.9%)
Other	6 (6.98%)
Laterality	
Left	46 (53.5%)
Right	40 (46.5%)
Tumor size(cm)	
IQR	7.2 [5.0; 9.3]
< 7	39 (45.3%)
≥ 7	47 (54.7%)
Grade stage	
Grade I	0 (0.0%)
Grade II	5 (7.8%)
Grade III	41 (64.1%)
Grade IV	18 (28.1%)
T stage	
T1	17 (20.5%)
T2	4 (4.82%)
T3	48 (57.8%)
T4	14 (16.9%)
N stage	
N0	30 (36.1%)
N +	53 (63.8%)
Surgery	
Yes	57 (66.3%)
None	29 (33.7%)
Radiotherapy	
Yes	20 (23.3%)
None/Unknown	66 (76.7%)
Chemotherapy	
Yes	45 (52.3%)
No/Unknown	41 (47.7%)

The median tumor size was 7.2 cm (IQR: 5.0–9.3 cm). Based on TNM staging system, 17 (20.5%), 4 (4.82%), 48 (57.8%), and 14 (16.9%) patients presented with T1, T2, T3 and T4 disease, respectively. 53(63.8%) patients showed lymph node metastasis. Tumor grading showed that 0 (0.0%), 5 (7.8%), 41 (64.1%), and 18 (28.1%) patients were at stages I, II, III and IV of the disease, respectively. In terms of treatment strategies, majority of the patients (66.3%) had undergone CNx, while remaining patients (33.7%) received non-surgical treatments. Moreover, 45 (52.3%) patients received chemotherapy, while 20 (23.3%) patients received radiotherapy.

#### Prognostic factors for OS and CSS

The median follow-up time was 5.0 months (3.0–10.8 months). The median OS and CSS time were 5.0 months (95% CI: 4.0–7.0) and 6 months (95% CI: 4.0–8.0), respectively. The OS rates at 1-, 2- and 5-years were 24.4%, 15.1% and 2.3%, respectively. Whereas, the CSS rates at 1-, 2- and 5-years were 27.0%, 17.9% and 2.8%, respectively.

**Figure 1:**
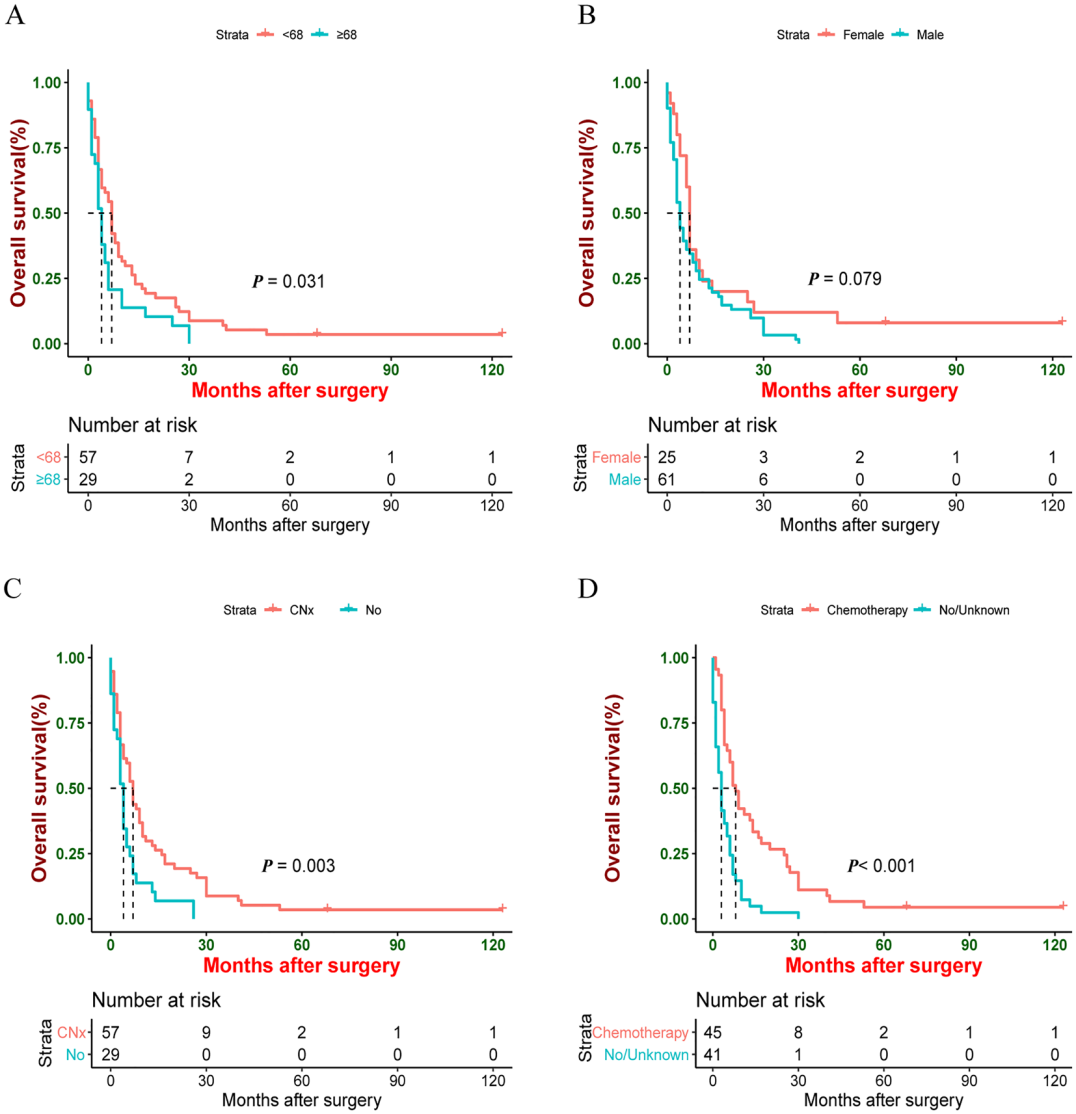
Kaplan–Meier estimate of Overall Survival (OS) by (**A**) Age, (**B**) Sex, (**C**) CNx, (**D**) Chemotherapy.

**Figure 2:**
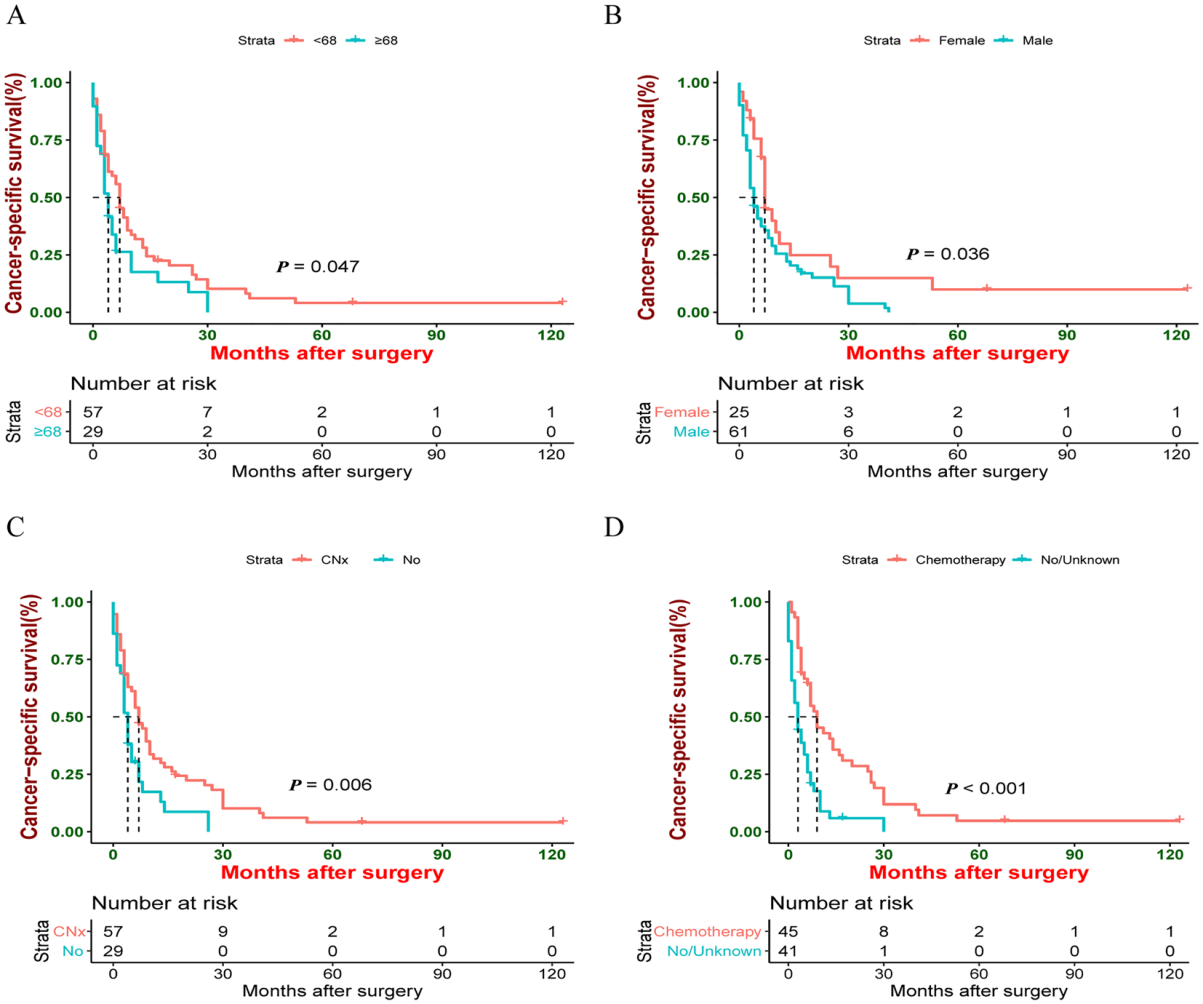
Kaplan–Meier estimate of Cancer-specific survival (CSS) by (**A**) Age, (**B**) Sex, (**C**) CNx, (**D**) Chemotherapy.

Older patients had worse OS and CSS than younger patients (median OS: 4 vs. 7 months, *P* = 0.031; median CSS: 4 vs. 7 months, *P* = 0.047) (Figs. 1A and 2A). Compared to male patients, female patients had better CSS (median OS: 7 vs. 4 months, *P* = 0.079; median CSS: 7 vs. 4 months, *P* = 0.036) (Figs. 1B and 2B). In terms of treatment modalities, patients receiving CNx exhibited better OS and CSS (median OS: 7 vs. 4 months, *P* = 0.003; median CSS: 7 vs. 4 months, *P* = 0.006) (Figs. 1C and 2C) compared to patients who did not undergo surgical treatment. Furthermore, OS and CSS of patients could benefit from chemotherapy (median OS: 8 vs. 3 months, *P* < 0.001; median CSS: 9 vs. 3 months, *P* < 0.001) (Figs. 1D and 2D). In contrast, OS and CSS were similar between patients undergoing radiotherapy and patients who did not receive radiotherapy (median OS: 6.5 vs. 6.5 months, *P* = 0.820; median CSS: 8 vs. 4.5 months, *P* = 0.490).

Univariate analysis of the factors that have an impact on OS and CSS of patients with metastatic CDC are presented in Tables 2 and 3. Using univariate Cox regression models, old age (OS: HR: 1.67, 95% CI: 1.06–2.65; *P* = 0.028; CSS: HR: 1.64, 95% CI: 1.02–2.63; *P* = 0.042), no-surgery (OS: HR: 2.02, 95% CI: 1.26–3.23; *P* = 0.004; CSS: HR: 1.98, 95% CI: 1.21–3.22; *P* = 0.006), and no-chemotherapy (OS: HR: 2.79, 95% CI: 1.76–4.42; *P* < 0.001; CSS: HR: 2.68, 95% CI: 1.67–4.31; *P* < 0.001) were identified as the potential risk factors that affect OS and CSS. Besides, male gender was another risk factor that was associated with CSS (CSS: HR: 1.75, 95% CI: 1.04–2.94; *P* = 0.037). All factors that had *P-*value ≤ 0.1 in univariate analysis were then enrolled into multivariate Cox regression model. Interestingly, only one factor that is, no-surgery was found to affect both, the OS and CSS (Tables 2 and 3).

**Table 2:** Univariate and Multivariate Cox Regression Analysis of the Associations between Clinicopathological Features and OS in Patients with Metastatic CDC.

Characteristics	Univariable analysis		Multivariable analysis	
	HR (95% CI)	*P*	HR (95% CI)	*P*
Age(years)				
< 68	Reference		Reference	
≥ 68	1.67 (1.06–2.65)	0.028	1.35 (0.83–2.20)	0.219
Sex				
Female	Reference			
Male	1.11 (0.81–1.52)	0.530		
Race				
Black	Reference			
White	1.05 (0.63–1.77)	0.841		
Other*	0.62 (0.25–1.55)	0.307		
Laterality				
Left	Reference			
Right	1.13 (0.73–1.73)	0.586		
Tumor size(cm)				
< 7	Reference			
≥ 7	1.38 (0.90–2.13)	0.144		
Pathological grade				
Grade II	Reference			
Grade III	1.24 (0.48–3.18)	0.660		
Grade IV	0.63 (0.23–1.76)	0.378		
T stage				
T1	Reference			
T2	1.47 (0.49–4.46)	0.493		
T3	0.91 (0.51–1.61)	0.743		
T4	1.13 (0.54–2.34)	0.745		
N stage				
N0	Reference			
N1	1.01 (0.60–1.70)	0.965		
N2	0.77 (0.44–1.33)	0.343		
Surgery				
Yes	Reference		Reference	
No	2.02 (1.26–3.23)	0.004	2.33 (1.45–3.77)	< 0.001
Radiotherapy				
Yes	Reference			
No/Unknown	1.05 (0.63–1.74)	0.862		
Chemotherapy				
Yes	Reference		Reference	
No/Unknown	2.79 (1.76–4.42)	< 0.001	2.79 (1.73–4.48)	< 0.001

CDC, renal collecting duct carcinoma; CI, confidence interval; HR, hazard ratio; OS, overall survival.

*Other included American Indian/Alaskan Native and Asian/Pacific Islander.

**Table 3:** Univariate and Multivariate Cox Regression Analysis of the Associations between Clinicopathological Features and CSS in Patients with Metastatic CDCs.

Characteristics	Univariable analysis		Multivariable analysis	
	HR (95% CI)	*P*	HR (95% CI)	*P*
Age (years)				
< 68	Reference		Reference	
≥ 68	1.64 (1.02–2.63)	0.042	1.26 (0.76–2.10)	0.368
Sex				
Female	Reference		Reference	
Male	1.75 (1.04–2.94)	0.037	1.39 (0.81–2.39)	0.232
Race				
Black	Reference			
White	1.22 (0.70–2.12)	0.491		
Other*	0.74 (0.29–1.91)	0.537		
Laterality				
Left	Reference			
Right	0.97 (0.62–1.52)	0.893		
Tumor size(cm)				
< 7	Reference			
≥ 7	1.35 (0.86–2.11)	0.187		
Pathological grade				
Grade II	Reference			
Grade III	1.52 (0.53–4.33)	0.434		
Grade IV	0.74 (0.24–2.28)	0.603		
T stage				
T1	Reference			
T2	1.05 (0.30–3.64)	0.941		
T3	0.87 (0.49–1.54)	0.637		
T4	1.05 (0.50–2.21)	0.894)		
N stage				
N0	Reference			
N1	0.93 (0.55–1.60)	0.803		
N2	0.79 (0.45–1.38)	0.412		
Surgery				
Yes	Reference		Reference	
No	1.98 (1.21–3.22)	0.006	2.23 (1.36–3.66)	0.001
Radiotherapy				
Yes	Reference			
No/Unknown	1.19 (0.69–2.06)	0.522		
Chemotherapy				
Yes	Reference		Reference	
No/Unknown	2.68 (1.67–4.31)	< 0.001	2.57 (1.56–4.23)	< 0.001

CDC, renal collecting duct carcinoma; CI, confidence interval; HR, hazard ratio; CSS, cancer-specific survival.

*Other included American Indian/Alaskan Native and Asian/Pacific Islander.

### Impact of surgery and chemotherapy on OS and CSS

**Figure 3:**
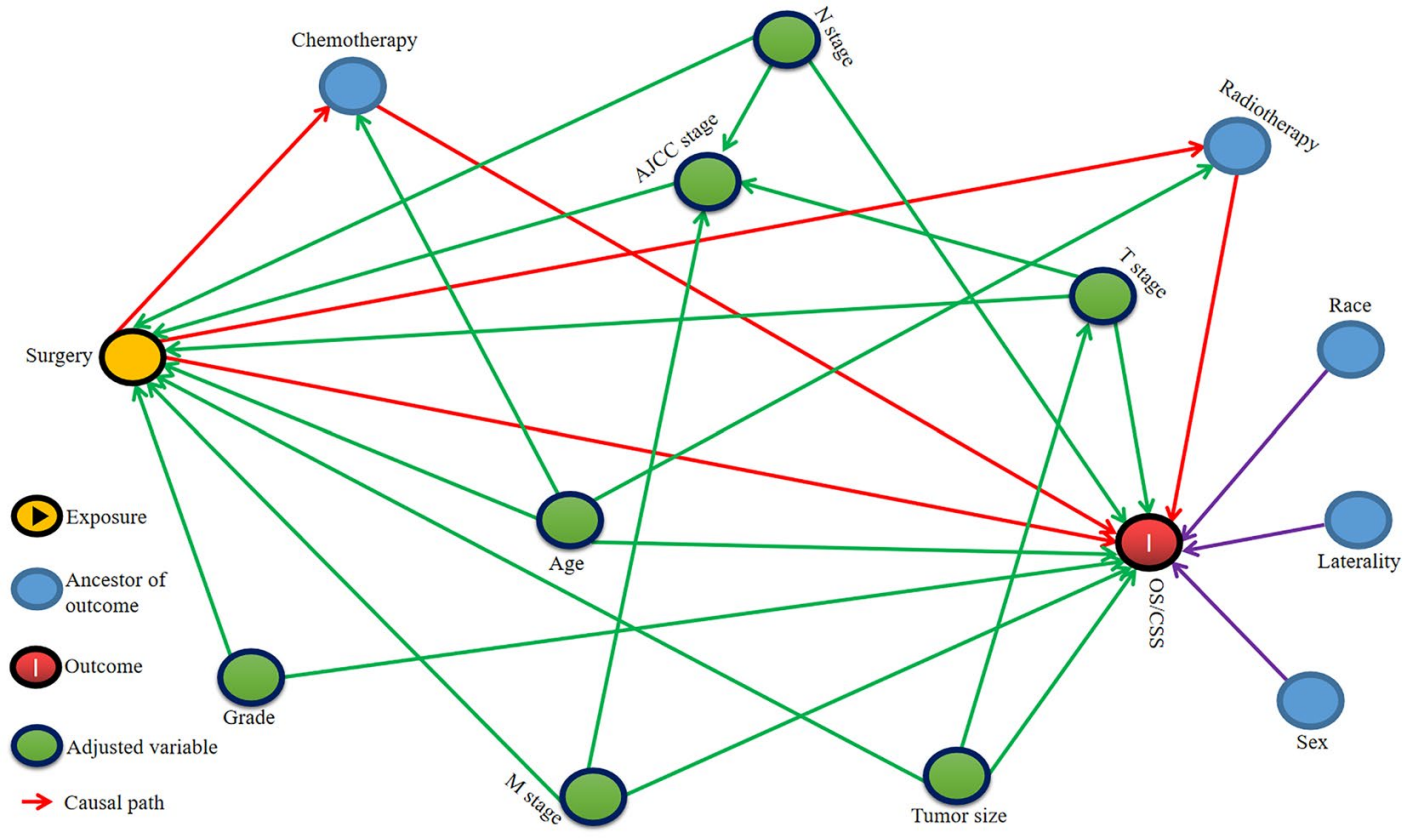
Directed Acyclic Graphs (DAG) show the impact of surgery on Overall Survival (OS) and Cancer-specific survival (CSS).

**Figure 4:**
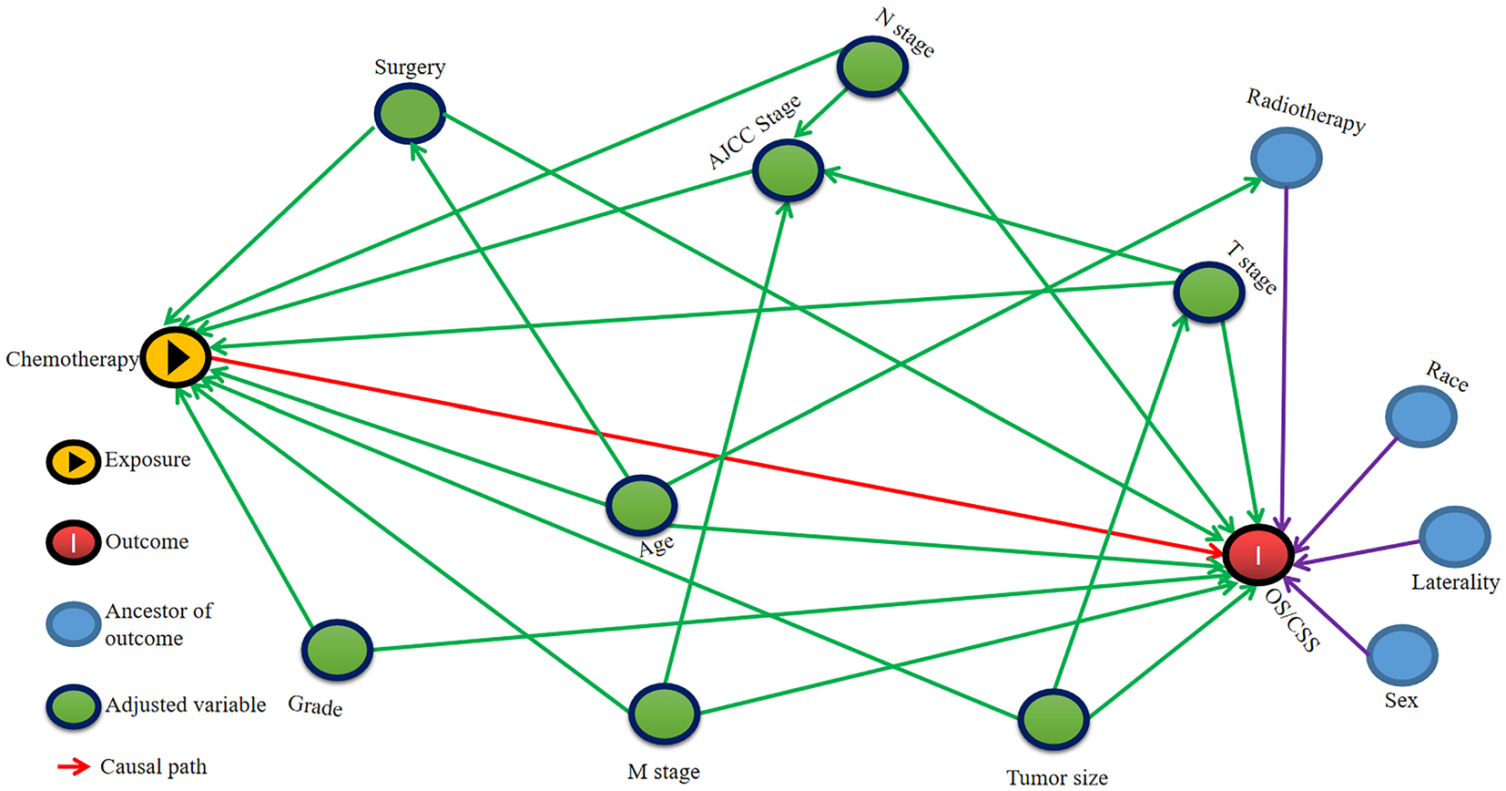
Directed Acyclic Graphs (DAG) exhibit the impact of chemotherapy on Overall Survival (OS) and Cancer-specific survival (CSS).

Given the interaction between variables, two DAGs were drawn to construct the multivariate Cox regression model. If surgery was considered as the main exposure factor, then chemotherapy and radiotherapy were excluded from the model as intermediate variables (Fig. 3). Then, in the multivariate model, surgery was associated with OS and CSS (OS: HR: 2.88, 95% CI: 1.20–6.99; *P* = 0.017; CSS: HR: 2.86, 95% CI: 1.16–7.08; *P* = 0.023) (Table 4). Similarly, when chemotherapy was chosen as the main exposure factor, surgery that wasn’t deemed as an intermediate variable, wasn't excluded (Fig. 4). In the second model, the association of chemotherapy with OS and CSS was verified (OS: HR: 3.78, 95% CI: 2.04–6.99; *P* < 0.001; CSS: HR: 3.32, 95% CI: 1.78–6.20; *P* < 0.001) (Table 5).

**Table 4:** DAG-Guided Multivariable Cox Regression Model Analysis for Causal Effect of Surgery on OS and CSS.

Variables	OS		CSS	
	HR (95% CI)	*P*	HR (95% CI)	*P*
Surgery				
Yes	Reference		Reference	
No	2.88 (1.20–6.88)	0.017	2.86 (1.16–7.08)	0.023
Age(years)				
< 68	Reference		Reference	
≥ 68	1.47 (0.80–2.71)	0.211	1.42 (0.76–2.66)	0.27
Tumor size(cm)				
< 7	Reference		Reference	
≥ 7	1.23 (0.65–2.30)	0.525	1.19 (0.63–2.26)	0.593
Pathological grade				
Grade II	Reference		Reference	
Grade III	1.16 (0.31–4.41)	0.824	1.20 (0.31–4.63)	0.786
Grade IV	0.63 (0.17–2.35)	0.487	0.61 (0.16–2.31)	0.464
T stage				
T1	Reference		Reference	
T2	1.49 (0.35–6.27)	0.587	1.05 (0.22–4.93)	0.953
T3	1.04 (0.36–2.95)	0.945	1.02 (0.36–2.95)	0.964
T4	0.93 (0.33–2.63)	0.893	0.88 (0.31–2.53)	0.812
N stage				
N0	Reference		Reference	
N1	0.81 (0.39–1.68)	0.570	0.74 (0.35–1.56)	0.428
N2	0.57 (0.28–1.17)	0.126	0.60 (0.29–1.23)	0.162

CDC, renal collecting duct carcinoma; DAG, directed acyclic graphs; CI, confidence interval; HR, hazard ratio; OS, overall survival; CSS, cancer-specific survival.

**Table 5:** DAG-Guided Multivariable Cox Regression Model Analysis for Causal Effects of Chemotherapy on OS and CSS.

Variables	OS		CSS	
	HR (95% CI)	*P*	HR (95% CI)	*P*
Chemotherapy				
Yes	Reference		Reference	
No/Unknown	3.78 (2.04–6.99)	< 0.001	3.32 (1.78–6.20)	< 0.001
Surgery				
Yes	Reference		Reference	
No	5.42 (2.07–14.17)	< 0.001	4.94 (1.84–13.22)	0.001
Age(years)				
< 68	Reference		Reference	
≥ 68	1.10 (0.59–2.06)	0.754	1.10 (0.58–2.09)	0.768
Tumor size (cm)				
< 7	Reference		Reference	
≥ 7	1.24 (0.64–2.41)	0.527	1.18 (0.60–2.32)	0.624
Pathological grade				
Grade II	Reference		Reference	
Grade III	0.91 (0.26–3.17)	0.885	0.97 (0.28–3.42)	0.962
Grade IV	0.58 (0.16–2.08)	0.406	0.58 (0.16–2.08)	0.401
T stage				
T1	Reference		Reference	
T2	2.51 (0.59–10.65)	0.213	1.63 (0.35–7.74)	0.536
T3	1.65 (0.57–4.83)	0.357	1.55 (0.53–4.55)	0.428
T4	1.10 (0.40–3.04)	0.847	1.02 (0.36–2.86)	0.967
N stage				
N0	Reference		Reference	
N1	0.87 (0.42–1.81)	0.706	0.78 (0.37–1.66)	0.523
N2	0.53 (0.25–1.13)	0.099	0.57 (0.27–1.21)	0.141

CDC, renal collecting duct carcinoma; DAG, directed acyclic graphs; CI, confidence interval; HR, hazard ratio; OS, overall survival; CSS, cancer-specific survival.

## Discussion

CDC patients, especially those with metastatic CDC, have poor prognosis and significantly lower survival rates than those with clear cell renal cell carcinoma. Recently, large cohort studies revealed that for CDC patients, CSS was similar to OS. This indicates that a vast majority of CDC patients eventually die due to CDC, further reflecting poor prognosis of CDC^4,7^. In the current study, the median OS and CSS time for CDC patients were 5 and 6 months, respectively. The OS rates at 1-, 2- and 5-years were 24.4%, 15.1% and 2.3%, respectively. Whereas, the CSS, rates at 1-, 2- and 5- years were 27.0%, 17.9% and 2.8%, respectively. Consistent to previous studies^4,11^, including patients with metastatic and non-metastatic CDC, which reported metastatic CDC had a worse prognosis, our study further identified that patients with metastatic CDC had extremely short survival time, and that most of the patients died due to CDC within 2 years of diagnosis. Thus, it is of great significance to undertake measures that improve the survival time of patients with metastatic CDC. Here, in this study, we described the relationship between surgery and prognosis in patients with metastatic CDC. Moreover, with the help of DAG-guided multivariable Cox regression model, we explained the role of CNx in the treatment of metastatic CDC, which might help clinicians to choose the appropriate treatment strategy.

The standard management for metastatic CDC remains inconsistent owing to the limited number of systematic studies focusing on it. Although CNx along with either chemotherapy or radiotherapy was used a common treatment modality for the managing metastatic CDC, the value of CNx management of patients with metastatic CDC has not yet been demonstrated. Sui et al. demonstrated that patients receiving CNx alone exhibited better survival time than those who did not receive CNx. Additionally, patients who received only surgery showed a reduced risk of death^6^. Moreover, findings revealed that patients with metastatic CDC undergoing both, CNx and chemotherapy/radiotherapy obtained maximum the most benefit. In addition, Abern and his colleagues showed that patients with metastatic CDC, who underwent surgery, had a better CSS than others, and that surgery could predict CSS^5^. Consistent with these findings, in the present study, we compared the survival differences between patients with metastatic CDC, receiving either CNx or non-surgical treatments. Additionally, we verified whether OS and CSS of patients could benefit from CNx. The difference in OS and CSS also existed in the DAG-guided multivariable Cox regression model analysis, indicating the significant role of CNx in improving survival. Therefore, if the patient is eligible for surgery, then CNx may be considered as a treatment for metastatic CDC.

CDC and urothelial carcinoma exhibit similar histological and clinical characteristics. Owing to this, some researchers suggest that chemotherapy agents that are effective for urothelial carcinoma may also benefit patients with metastatic CDC^1,12^ Tokuda et al. and Motzer et al. found that patients with metastatic CDC could exhibit a partial response to gemcitabine and either cisplatin or carboplatin therapy^8,13^. Subsequently, patients with metastatic CDC who received gemcitabine along with either cisplatin or carboplatin therapy showed a 26% partial or complete response rate. This included one complete response in a prospective phase II trial as reported by Oudard et al.^10^ Lately, Pecuchet et al. demonstrated that patients receiving a combination of bevacizumab and platinum-based chemotherapy have longer OS than those receiving platinum-based chemotherapy alone. These findings are consistent with the results of a previous clinical trial, including five untreated patients with metastatic CDC. Of these, three patients exhibited a partial response, one had stable disease, and one achieved complete remission after CNx of the metastatic site^14^. Tumor progression and first-line treatment failure are the common problems associated with metastatic CDC. Similarly, specific regimen of chemotherapy was not provided, which is the defect of the data used in this study. Chemotherapy increased the survival of patients with metastatic CDC, and was associated with OS and CSS in the DAG-guided multivariable Cox regression model, after the exclusion of irrelevant mediation variables. Although Wilson S et al. verified that radiotherapy plays a certain role in preventing the progression of the disease^6^, the benefits of radiotherapy for managing metastatic CDCs has not been discovered in other studies^8,10,15^. In all, patients with metastatic CDC can benefit from a chemotherapy but not radiotherapy. However, prospective clinical studies are needed to confirm the feasibility of the standard chemotherapy for treating metastatic CDC.

Certainly, there exist several limitations in the current study, which should be acknowledged. Firstly, median survival of all patients was 5 months and their 1-, 2-,5-years survival rates were very short, which was caused by the dismal prognosis. Therefore, the impact of OS/CCS on 1-, 2-,5-years survival rates was limited. The endpoint of CCS is not optimal prognostic indicators, since the quality of assessment for this endpoint strongly varies and is shaky. Recurrence free survival due to mostly limited diseases stages would be more reliable endpoints for the analysis, which most likely was not available within the SEER database. Secondly, the data we used was derived from the SEER database. The present study included only a small number of patients and was retrospective in nature. Therefore, potential selection bias was inevitable in this study. Thirdly, because the specific chemotherapy regimens were unclear and the response of patients was not record, the specific effects of these agents on metastatic CDC may be limited. Fourly, some patients received targeted therapeutic agents with minimal toxicity, including sorafenib, sunitinib and temsirolimus, and showed a good response^14,16,17^. For data lacking information on targeted therapeutics, the impact of it on the outcome of metastatic CDC could not be determined. Finally, a centralized pathological review was not performed, and CDC was easily misdiagnosed as others, such as medullary carcinoma and FH-deficient RCC, due to their similar microscopic features. Despite these limitations, this study can help clinicians to evaluate the prognosis of metastatic CDC and select the appropriate treatment for this disease.

In conclusion, metastatic CDC is an extremely rare renal carcinoma and exhibits poor survival time. This study identified that patients with younger age, receiving CNx and chemotherapy exhibit better survival outcomes. The multivariable regression model identified non-surgical treatment as the only prognostic factor for OS and CSS. However, the DAG-guided multivariate Cox regression model suggested that CNx and Chemotherapy were associated with OS and CSS. Conclusively, CNx and chemotherapy can be applied for the management of metastatic CDCs. However, long-term large-scale prospective trials are required to validate these results.

## Methods

### Patient population and selection criteria

SEER*Stat 8.3.9.2 software (https://seer.cancer.gov/seerstat/) was used to retrieve information of patients, who had a confirmed diagnosis of CDC between 2000 and 2018, from the SEER database. SEER database is a program launched by the National Cancer Institute, and includes cancer information on tumor features, demographic factors, initial treatment modalities and outcomes of approximately 35% of the entire U.S. population^18,19^. Information of all patients was obtained through a public method, and hence ethical approval was not required. The “Incidence-SEER Research Plus Data, 18 Registries, Nov 2020 Sub (2000–2018)” database was selected. As per the 3rd edition of the International Classification of Diseases for Oncology (ICD-O-3), data of morphology codes 8319/3 (collecting duct carcinoma) and 8319/2 (collecting duct carcinoma in situ), were extracted. The inclusion criteria were as follows: (1) complete survival information of patients was available; (2) diagnosis of CDC was verified through histological examination; (3) unilateral tumor in kidney; (4) patients had complete information on TNM stage and pathological grade. Eventually, from the 521 patients with CDC, 86 patients with distant metastasis were recruited in this study. The study was conducted in accordance with the relevant guidelines and regulations.

### Study variables

The following patient information was chosen and exported. Patients’ demographic characteristics included age, race, sex, living status, and survival time. Tumor characteristics consisted of laterality, grade, American Joint Committee on Cancer (AJCC) stage and Tumor Node Metastasis (TNM) classification. Moreover, details on treatment strategies for CDCs, including surgery, chemotherapy, and radiotherapy, were obtained. Some patients received chemotherapy or radiotherapy for palliation. The specific schedule and the site of radiotherapy are unclear. Overall survival (OS) and cancer-specific survival (CSS) were deemed as the primary and secondary endpoints, respectively. OS was defined as the time from the date of first confirmed diagnosis to the date of death by any cause or last follow-up. CSS was defined as the time from the date of first confirmed diagnosis to the date of death caused by metastatic CDC.

### Statistical analysis

All statistical tests were conducted using R-3.6.3 software (The R Foundation for Statistical Computing, Vienna, Austria). The receiver operating characteristic (ROC) curve was constructed to obtain the optimal cut-off value for age, which was 68 years in this study. Descriptive categorical variables were described in numbers and percentages during descriptive statistics. Median survival time and survival analysis were performed using the Kaplan–Meier method. The log-rank test was used for comparison of survival differences among enumeration data. Cox regression analysis was applied to recognize the factors affecting OS and CSS. Based on the different DAGs, two multivariable regression models were constructed to demonstrate the effect of surgery and chemotherapy on outcomes respectively^20,21^. Comparison among groups was considered to be statistically significant if *P *values were < 0.05(for two-sided test).

### Ethics approval

Since the data obtained from the SEER registry are de-identified and public, institutional review board approval can be waived and informed consent is not required for this study.

## Supplementary Information

Supplementary Information.

## Data Availability

All raw data presented in this study can be obtained from the SEER database Official website (https://seer.cancer.gov/).

## Abbreviations

CDC: Collecting duct carcinoma
SEER: Surveillance, Epidemiology, and End Results
OS: Overall survival
CSS: Cancer-specific survival
CNx: Cytoreductive nephrectomy
DAG: Directed acyclic graphs
IQR: Interquartile range
HR: Hazard ratio
CI: Confidence interval
Cox: Proportional hazards regression model
TNM: Tumor Node Metastasis
